# Microvesicles derived from hypoxia/reoxygenation-treated human umbilical vein endothelial cells promote apoptosis and oxidative stress in H9c2 cardiomyocytes

**DOI:** 10.1186/s12860-016-0100-1

**Published:** 2016-06-23

**Authors:** Qi Zhang, Man Shang, Mengxiao Zhang, Yao Wang, Yan Chen, Yanna Wu, Minglin Liu, Junqiu Song, Yanxia Liu

**Affiliations:** Department of Pharmacology, School of Basic Medical Sciences, Tianjin Medical University, No. 22, Qixiangtai Road, Heping District, Tianjin, 300070 People’s Republic of China; Section of Endocrinology, Department of Medicine, Temple University School of Medicine, 3500 North Broad Street, Room 480A, Philadelphia, PA 19140 USA; Department of Dermatology, Perelman School of Medicine, University of Pennsylvania, Philadelphia, PA, 19104 USA

**Keywords:** Endothelial microvesicles, Oxidative stress, Apoptosis, H9c2 cardiomyocytes, Hypoxia/reoxygenation

## Abstract

**Background:**

Vascular endothelial dysfunction is the closely related determinant of ischemic heart disease (IHD). Endothelial dysfunction and ischemia/reperfusion injury (IRI) have been associated with an increase in microvesicles (MVs) in vivo. However, the potential contribution of endothelial microvesicles (EMVs) to myocardial damage is unclear. Here we aimed to investigate the role of EMVs derived from hypoxia/reoxygenation (H/R) -treated human umbilical vein endothelial cells (HUVECs) on cultured H9c2 cardiomyocytes.

**Results:**

H/R injury model was established to induce HUVECs to release H/R-EMVs. The H/R-EMVs from HUVECs were isolated from the conditioned culture medium and characterized. H9c2 cardiomyocytes were then incubated with 10, 30, 60 μg/mL H/R-EMVs for 6 h. We found that H9c2 cells treated by H/R-EMVs exhibited reduced cell viability, increased cell apoptosis and reactive oxygen species (ROS) production. Moreover mechanism studies demonstrated that H/R-EMVs could induce the phosphorylation of p38 and JNK1/2 in H9c2 cells in a dose-dependent manner. In addition, H/R-EMVs contained significantly higher level of ROS than EMVs generated from untreated HUVECs, which might be a direct source to trigger a cascade of myocardial damage.

**Conclusion:**

We showed that EMVs released during H/R injury are pro-apoptotic, pro-oxidative and directly pathogenic to cardiomyocytes in vitro. EMVs carry ROS and they may impair myocardium by promoting apoptosis and oxidative stress. These findings provide new insights into the pathogenesis of IRI.

## Background

Ischemic heart disease (IHD) is the major cause of death worldwide. The pathological processes leading to IHD (including myocardial infarction, angina pectoris, or both) are very complicated and closely accompanied with ischemia/reperfusion injury (IRI) [1]. It is generally accepted that oxidative stress is responsible for the damage of IRI, which is often associated with vascular dysfunction [2]. The endothelial cells that line the inner layer of blood vessels form a vital and dynamic structure that is essential for vascular hemostatic balance. These cells appear to be particularly vulnerable to the deleterious effects of both hypoxia (ischemia) and reoxygenation (reperfusion) [3].

Microvesicles (MVs) are small vesicles of 0.1 ~ 1 μm diameter released from stimulated or apoptotic cells, such as platelets, endothelial cells, lymphocytes, erythrocytes and even smooth muscle cells [4]. MVs contain a subset of cell surface proteins derived from the plasma membrane of the original cells, which allow them to function as messengers that mediate many biological processes [5, 6]. In addition, MVs also carry various bioactive molecules, such as cytokines, RNA and DNA derived from their metrocyte, which can be transferred into target cells and mediate a series of biological effects [7–9]. Increased levels of circulating MVs have been suggested in acute coronary ischemia, myocardial infarction and other IHD, and MVs are likely contributing to endothelial dysfunction, leukocyte adhesion, platelet activation and obstruction of blood flow [10].

It has been reported that endothelial microvesicles (EMVs) may participate in inflammatory responses or angiogenesis, and propagate biological responses involved in haemostatic balance [11, 12]. Recent evidence suggests that EMVs may contribute to the oxidative injury and cell apoptosis in the course of IRI [13]. EMVs derived under pathological high glucose conditions induce adhesion protein expression in endothelial cells and subsequent monocyte adhesion in a NADPH oxidase-ROS-p38-dependent way [14]. Our group previously reported that MVs derived from hypoxia/reoxygenation-treated HUVECs impaired relaxation of rat thoracic aortic rings, and declined the production of NO and the expression of p-eNOS [15]. In this experiment, we established hypoxia/reoxygenation injury model to induce EMVs release in vitro and investigated its role on endothelial function of the aortic rings. However, the detailed mechanisms underlying EMVs-mediated cardiac damage and its relation to oxidative stress are not clear. Here we demonstrated the pathogenic roles of H/R-EMVs: (i) to cause cardiomyocytes injury directly; (ii) to promote cardiomyocytes apoptosis; (iii) to generate ROS in cardiomyocytes.

## Methods

### Cell culture

Human umbilical vein endothelial cells (HUVECs, Human EA.hy926 endothelial cells, Cell bank of Chinese Academy of Sciences, Shanghai, China) and H9c2 cells (ATCC, Manassas, VA, USA) were cultured in DMEM (Hyclone, Logan, UT, USA) with 10 % FBS (Gibco, CA, USA) under standard cell-culture conditions (37 °C, 5 % CO_2_). All procedures were performed in accordance with the Declaration of Helsinki of the World Medical Association and the research protocol was approved by Ethics Committee of Tianjin Medical University.

### H/R-EMVs preparation

To generate endothelial microvesicles (EMVs), HUVECs were stimulated by hypoxia/reoxygenation (H/R) as previously described [15]. HUVECs of passage 5–8 were used when 70–80 % confluent. Briefly, HUVECs were subjected to hypoxic buffer (in mM: 0.9 NaH_2_PO_4_, 6.0 NaHCO_3_, 1.0 CaCl_2_, 1.2 MgSO_4_, 20.0 HEPES, 98.5 NaCl, 10.0 KCl, 40.0 sodium lactate, pH 6.2) in a hypoxic chamber (95 % N_2_ and 5 % CO_2_, Billups-Rothenberg, Del Mar, CA, USA) for 12 h and then reoxygenated under standard cell-culture conditions for 4 h. Hypoxic buffer was collected in 15-mL centrifuge tubes and centrifuged at 2 700 g, 4 °C for 20 min to remove cell debris. Most supernatants were collected in 13.2-mL ultracentrifuge tubes and centrifuged at 33 000 rpm for 150 min to pellet H/R-EMVs. The pellet was resuspended in 100 μL PBS and kept at −20 °C.

### H/R-EMVs characterization by flow cytometry

H/R-EMVs were characterized by flow cytometry in terms of size assessment and biomarker identification. After centrifuging at 2 700 g, 4 °C for 20 min, aliquots of 90 μL supernatant without cell debris were collected and fixed with paraformaldehyde (PFA, Boster immunoleader, Wuhan, China) to a final concentration of 1 % for 1 h at room temperature (RT), then snap-frozen in liquid nitrogen and stored at −80 °C. For flow cytometry analysis, 10 μL fixed cell-free supernatants were blocked with mouse serum (Zhongkechenyu, Beijing, China) and then incubated with 5 μL anti-PE-CD144 antibody or its anti-PE Mouse IgG1 isotype (Santa Cruz, CA, USA) in dark for 30 min at RT, respectively. Latex beads of 1 μm (Molecular Probe, Invitrogen, Carlsbab, CA, USA) were used to calibrate gain setting and evaluate the size of EMVs. Events < 1 μm in diameter and CD144 positive were defined as H/R-EMVs. Each sample was analyzed with the flow cytometer (FACS Calibur, BD biosciences, Franklin Lakes, NJ, USA). Protein quantification of H/R-EMVs was performed by a BCA protein assay (Beyotime, Nanjing, China).

### Treatment with H/R-EMVs on H9c2 cells

H9c2 cells of passage 6–10 were used when 70–80 % confluent. For subsequent experiments, H9c2 cells were incubated with 10, 30, 60 μg/mL H/R-EMVs for 6 h. After H/R-EMVs treatment, culture supernatants and protein extracts of H9c2 cells were collected for further study.

### Colorimetric assay of cell viability and LDH activity

Cell viability was determined using methyl thiazolyl tetrazolium (MTT, Amresco, Solon, OH, USA) method. H9c2 cells cultured in 96-well plates at 1 × 10^5^ cells/mL were treated with H/R-EMVs for 6 h. Then they were incubated with 10 μL 0.5 % MTT solution for 4 h at 37 °C. The supernatant was discarded after the incubation and 150 μL dimethyl sulfoxide was added to each well. The culture plate was shaken at high speed for 10 min until crystals dissolved completely. The absorbance of the blue formazan derivative was measured at a wavelength of 490 nm using a microplate reader (Bio-Rad Laboratories, CA, USA).

Lactate dehydrogenase (LDH) release detection was performed using a LDH Kit (Jiancheng Bioengineering Institute, Nanjing, China). Culture supernatants of H9c2 cells were collected after 6 h incubation with H/R-EMVs. Each supernatant (20 μL) was transferred to a fresh 96-well plate and an equal volume of freshly prepared reaction mixture was added according to the manufacturer’s instruction. The absorbance was measured at a wavelength of 450 nm using the microplate reader following 15 min incubation at 37 °C. All experiments were repeated three times independently.

### H9c2 cell apoptosis assay

Apoptosis of H9c2 cells was examined by Hoechst 33258 staining, flow cytometry with Annexin V-FITC/PI staining and caspase 3 activity. After H/R-EMVs treatment, H9c2 cells in 6-well plates were washed twice with PBS and stained with 10 μg/mL Hoechst 33258 (KeyGen Biotech, Nanjing, China) at 37 °C for 20 min, and then examined under a fluorescent microscope (Nikon Melville, NY, USA) with the excitation wavelength of 350 nm for morphological changes.

To perform a quantitative analysis of cell apoptosis, flow cytometry with Annexin V-FITC/PI staining was employed. H9c2 cells were incubated with 5 μL Annexin V-FITC and PI (BD biosciences, Franklin Lakes, NJ, USA) for 15 min at RT in dark. Cells from each sample were then analyzed by FacsCalibur flow cytometer. The data was analyzed using Flowjo software.

For detection of the activity of caspase 3, H9c2 cells in 6-well plates were trypsinized and collected, then lysed at 4 °C for 15 min in a caspase 3 lysis buffer (Beyotime, Nanjing, China). Protein extracts of 10 μL were incubated with 90 μL reaction buffer containing 2 mM caspase-3 substrate (Ac-DEVD-pNA) for 2 h at 37 °C. The absorbance was measured at a wavelength of 405 nm using a multilabel reader (Bio-Tek, Winooski, VT, USA). Results were expressed as nmol/μg protein.

### Determination of lipid peroxidation level and superoxide dismutase activity

Lipid peroxidation levels in H9c2 cells were determined by estimating malondialdehvde (MDA) levels using the thiobarbituric acid reactive substance (TBARS) test (Jiancheng Bioengineering Institute, Nanjing, China). Cells were lysed by 1 % Triton-X 100 for 30 min on ice and then centrifuged at 12 000 g, 4 °C for 10 min. Protein concentration of the supernatants was determined by the BCA protein assay. Aliquots of 30 μL supernatants were incubated with reactive solutions according to the product instructions. The supernatant absorbance was measured at a wavelength of 532 nm. The results were expressed as nmol/mg protein.

The activity of superoxide dismutase (SOD) was measured in terms of inhibition of superoxide anions. Protein samples were prepared in the same way as MDA assay. Aliquots of 30 μL supernatants were incubated with reactive solutions at 37 °C water bath for 40 min. The absorbance was measured at a wavelength of 550 nm. SOD activities (U/mg protein) were calculated using the equation provided by the manufacture (Jiancheng Bioengineering Institute, Nanjing, China).

### Measurement of reactive oxygen species

Both reactive oxygen species (ROS) content in H/R-EMVs and ROS production in H9c2 cells were determined by 2′,7′-dichlorodihydrofluorescein diacetate assay (DCFH-DA, Beyotime, Nanjing, China). Pelleted H/R-EMVs and adherent MVs-treated H9c2 cells were diluted with 10 μM DCFH-DA and incubated for 20 min at 37 °C in dark, respectively. DCF intensity of MVs samples was analyzed with flow cytometry. To measure cellular ROS production, some MVs-treated H9c2 cells were washed and observed using fluorescent microscopy with the excitation wavelength of 520 nm. The other MVs-treated H9c2 cells were washed, trypsinized, pelleted and resuspended with PBS at 1 × 10^6^ cells/mL. DCF intensity of these cell samples was also measured using flow cytometry.

### Western blot analysis of Bcl-2/Bax, p-p38 and p-JNK1/2

H9c2 cells were lysed in a lysis buffer (20 mM Tris pH 7.5, 150 mM NaCl, 1 % Triton X-100, sodium pyrophosphate, β-glycerophosphate, EDTA, Na_3_VO_4_, leupeptin, Beyotime, Nanjing, China) at 4 °C for 30 min. Protein concentration was measured using BCA assay. Equal amounts of proteins (80 μg) were loaded into 12 % SDS electrophoresis and transferred onto PVDF membranes. Blots were incubated with blocking buffer for 60 min at RT, then incubated with the relevant primary antibodies (anti-β-actin, anti-Bcl-2, anti-Bax, anti-p-JNK1/2 antibody, Santa Cruz, CA, USA; anti-p-p38 MAPK, anti-p38 MAPK, anti-JNK1/2 antibody, Cell Signaling Technology, Danvers, MA, USA) overnight at 4 °C, followed by the corresponding HRP-conjugated secondary antibodies for 120 min. Then proteins were revealed by chemiluminescence using the ECL kit (Beyotime, Nanjing, China).

### Statistical analysis

Data were expressed as mean ± standard derivation (SD). The one-way analysis of variance (ANOVA) was used for multiple comparisons. Statistical evaluation was performed using GraphPad Prism 5. The value of *P <* 0.05 was considered statistically significant. All experiments were repeated three times independently.

## Results

### Characterization of H/R-EMVs

**Fig. 1:**
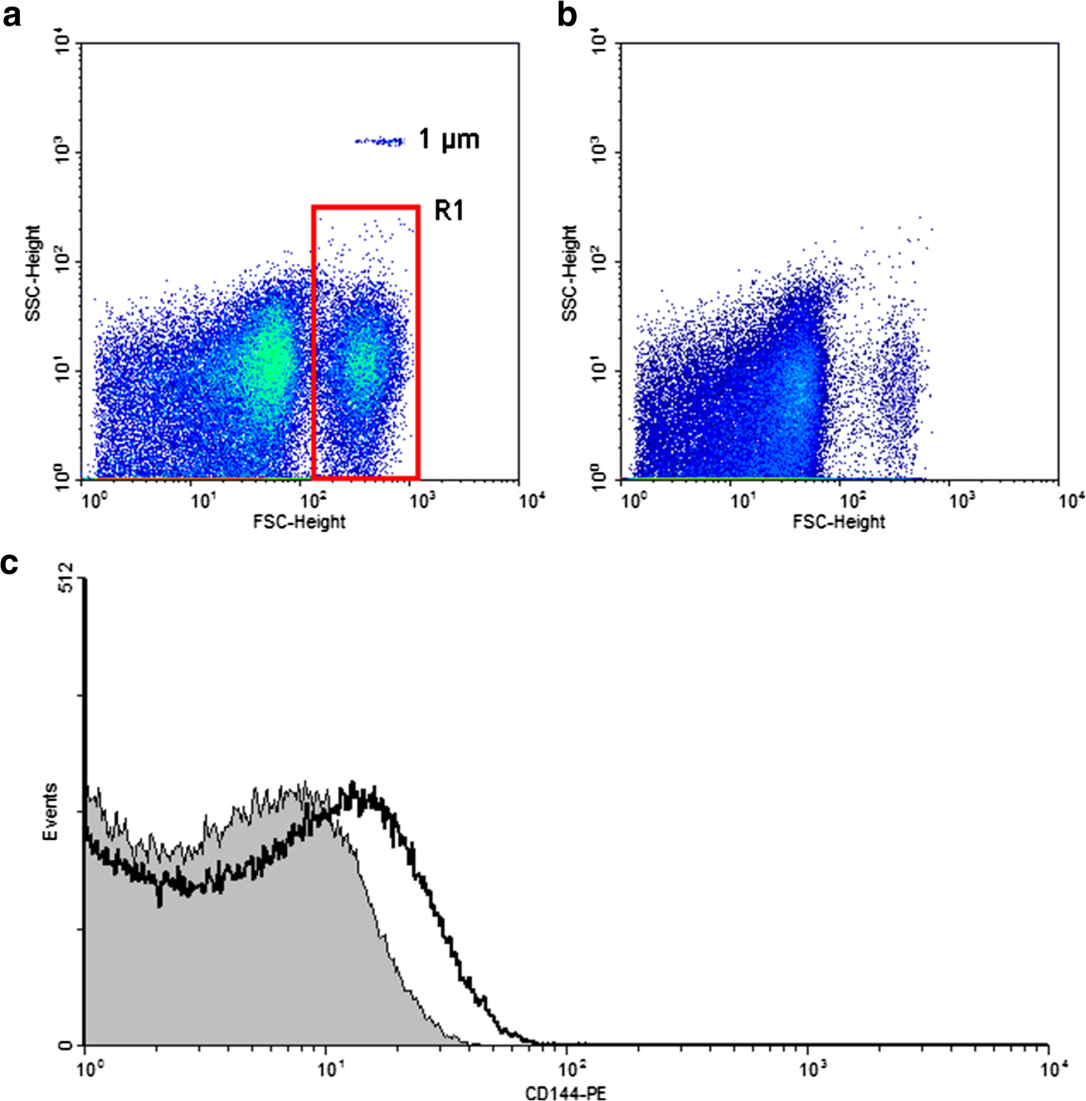
Flow cytometric analysis of H/R-EMVs. **a** Representative dot plots for H/R-EMVs. H/R-EMVs were gated in R1 which below the area of 1 μm-calibration beads. **b** Representative dot plots of FSC versus SSC for evaluation of the vehicle of H/R-EMVs. **c** Representative histogram for CD144^+^ H/R-EMVs. The solid gray histogram corresponds to H/R-EMVs with isotype antibody. The open black histogram corresponds to H/R-EMVs with anti-PE-CD144 antibody

H/R stimulation of HUVECs resulted in the formation of MVs of about <1 μm in diameter as assessed by flow cytometry. HUVECs were first exposed to hypoxia (12 h) and reoxygenation (4 h). The H/R injury decreased HUVECs viability to 70.53 ± 2.61 % compared with control (*P* < 0.001, supplemental data). Flow cytometry analysis of H/R-EMVs was used to determine their size and cellular origin. Using 1 μm beads as size standards, the majority of H/R-EMVs were observed around the forward scatter signal corresponding to 1 μm beads (Fig. 1a, b). Cellular origin was identified by investigating the specific surface antigens of MVs. Most of H/R-EMVs externalized their endothelial cell marker CD144 (Fig. 1c). These results indicated that H/R-EMVs had a size of <1 μm and expressed on their surface adhesion molecules of HUVECs from which they originated.

### H/R-EMVs reduced the viability of H9c2 cells

**Fig. 2:**
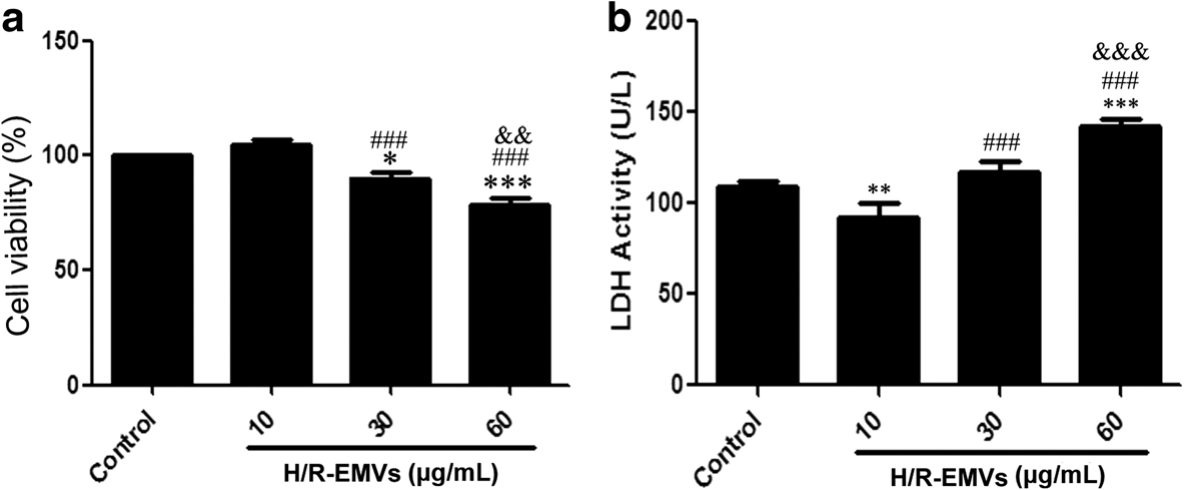
H/R-EMVs exerted cytotoxic effect on H9c2 cells. **a** MTT assays. H9c2 cells were treated with 10, 30, 60 μg/mL H/R-EMVs for 6 h. The viability of H9c2 cells was expressed as a percentage relative to non-H/R-EMVs-treated control cells. Control cells were considered to be 100 % viable. **b** LDH activity assay. H9c2 cells were treated at the concentration of 10, 30, 60 μg/mL H/R-EMVs for 6 h to detect LDH activity. *p < 0.05, ***p < 0.001 versus control; ^###^ *p* < 0.001 versus H/R-EMVs 10 μg/mL; ^&&^ *p* < 0.01, ^&&&^ *p* < 0.001 versus H/R-EMVs 30 μg/mL

To explore whether H/R-EMVs influence the progression of myocardium damage, target H9c2 cardiomyocytes were exposed to 10, 30, 60 μg/mL H/R-EMVs for 6 h, respectively. Compared with control group, 10 μg/mL H/R-EMVs showed little effect on H9c2 cells, whereas 30 and 60 μg/mL H/R-EMVs significantly decreased H9c2 cell viability by 10 % and 20 %, respectively (*P* < 0.05, Fig. 2a). The cytotoxic effect of H/R-EMVs was further confirmed using LDH assay. H/R-EMVs of 10 μg/mL did not induce more LDH leakage than the control group. As expected, H9c2 cells exposed to 30 and 60 μg/mL H/R-EMVs exhibited more release of LDH through damaged cell membranes (*P* < 0.05, Fig. 2b). These findings indicated that H/R-EMVs displayed a dose-dependent cytotoxicity in H9c2 cardiomyocytes.

### H/R-EMVs increased H9c2 cell apoptosis

**Fig. 3:**
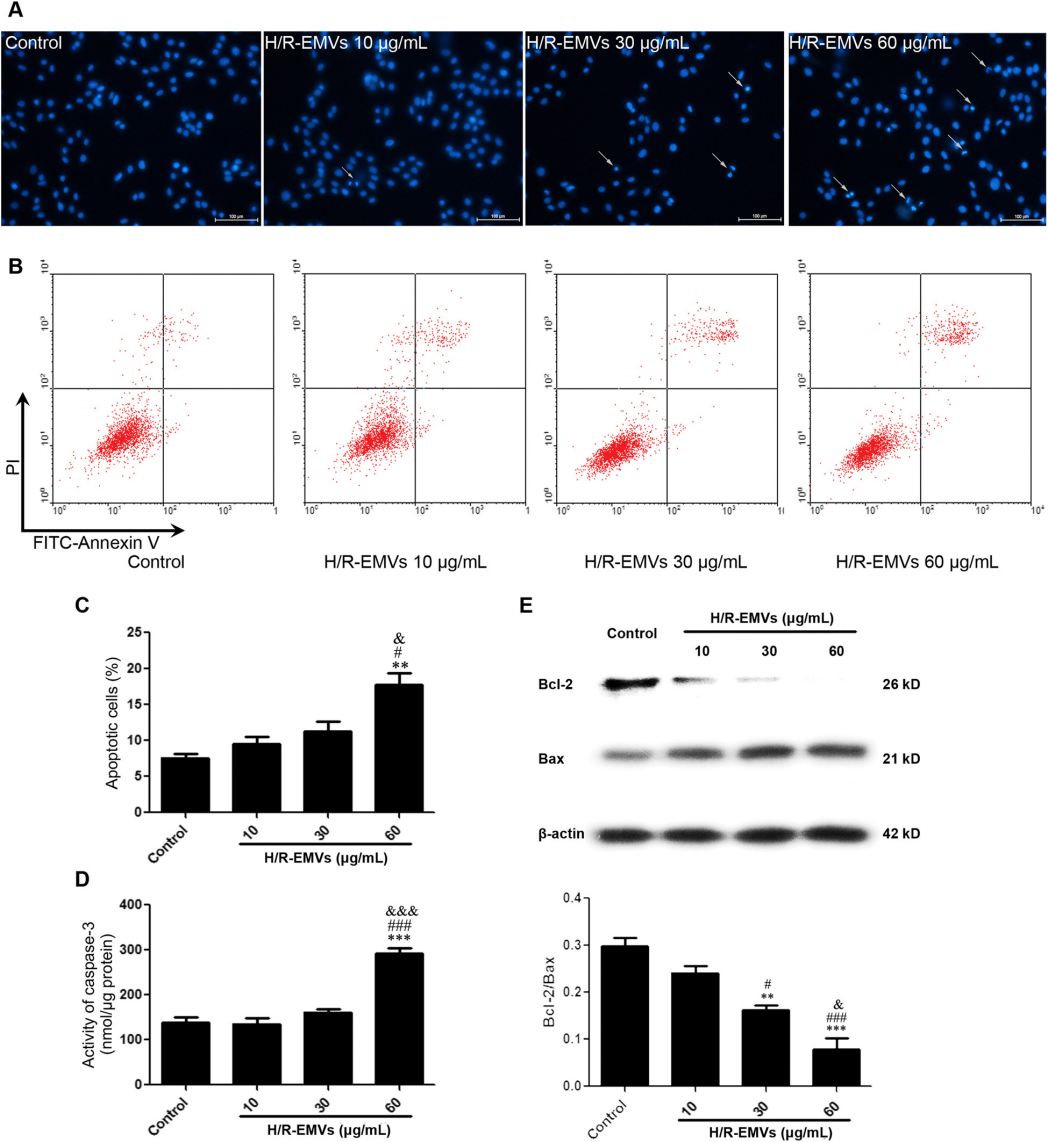
H/R-EMVs exhibited pro-apoptotic effect on H9c2 cells. **a** Hoechst 33258 staining was used to observe the apoptotic cells after treatment with 10, 30, 60 μg/mL H/R-EMVs for 6 h (100×). The arrows show apoptotic cells. The untreated cells serve as Control. **b** Annexin V-FITC/PI double staining. The results were interpreted in the following fashion: cells in the lower-left quadrant (Annexin V−/PI−) represent living cells; those in the lower-right quadrant (Annexin V+/PI−) represent early apoptotic cells; those in the upper-right quadrant (Annexin V+/PI+) represent late apoptotic cells; and those in the upper-left quadrant (Annexin V−/PI+) represent necrotic cells. **c** The apoptotic rate of H9c2 cells was quantified according to flow cytometry analysis. **d** Caspase 3 activity of H9c2 cells was measured after treatment with 10, 30, 60 μg/mL H/R-EMVs for 6 h. **e** Representative images of immunoblots with antibodies against Bcl-2, Bax, β-actin, respectively among groups. β-actin serves as a loading control. ^*^ *p* < 0.05, ^**^ *p* < 0.01, ^***^ *p* < 0.001 versus Control; ^#^ *p* < 0.05, ^##^ *p* < 0.01, ^###^ *p* < 0.001 versus H/R-EMVs 10 μg/mL; ^&^ *p* < 0.05, ^&&&^ *p* < 0.001 versus H/R-EMVs 30 μg/mL

In order to investigate the mechanisms of H/R-EMVs-induced cell death, we performed Hoechst 33258 staining and Annexin V-FITC/PI staining. After being treated with H/R-EMVs for 6 h, H9c2 cells were stained with Hoechst 33258 and observed under the fluorescence microscope. The dye stains condense chromatin of apoptotic cells more brightly than that of normal cells. H/R-EMVs groups in the concentrations of 10 and 30 μg/mL showed few fragmented nuclei, while 60 μg/mL H/R-EMVs group displayed apparently increasing number of fragmented or condensed nuclei (Fig. 3a). Flow cytometry with Annexin V-FITC/PI staining showed 60 μg/mL H/R-EMVs increased the apoptotic rate of target H9c2 cells by 18 % (*P* < 0.05, Fig. 3b, c).

To confirm the co-incubation of H9c2 cells with high dose of H/R-EMVs is contributable to their apoptosis, the activity of caspase 3 and expression of Bcl-2/Bax in target cells were determined. Consistent with the rates of Annexin V positive cells, caspase 3 activity of H9c2 cells stimulated by 60 μg/mL H/R-EMVs increased to 294.14 ± 28.03 nmol/μg, compared with 140.23 ± 29.43 nmol/μg in control group (*P* < 0.05, Fig. 3d). In addition, high dose (60 μg/mL) of H/R-EMVs also induced higher activity of caspase 3 than 10 and 30 μg/mL H/R-EMVs (*P* < 0.05, Fig. 3d). The dynamic balance of Bcl-2 and Bax determines a cell’s fate. Bax levels increase but Bcl-2 levels decrease during cell apoptosis. Western blot analysis revealed that Bcl-2/Bax ratio in H9c2 cells gradually decreased with the increasing dose of H/R-EMVs (Fig. 3e). Therefore, the pro-apoptotic effect of H/R-EMVs in H9c2 cells was associated with Bcl-2 inhibition and Bax up- regulation.

### H/R-EMVs induced the oxidative damage in H9c2 cells by MDA and ROS production

**Fig. 4:**
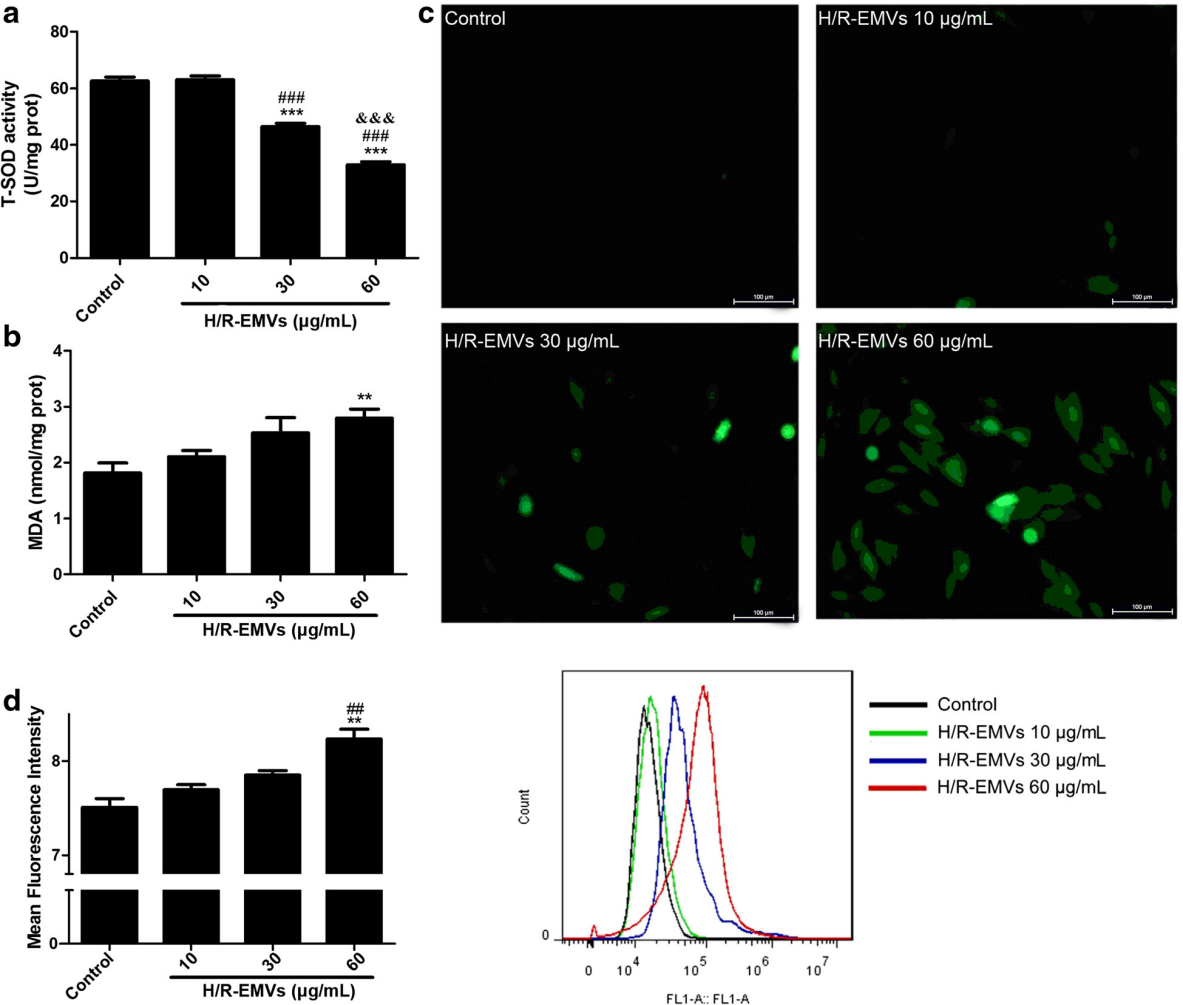
H/R-EMVs induced the oxidative damage in H9c2 cells. **a** The activity of SOD in H9c2 cells was examined after being treated with 10, 30, 60 μg/mL H/R-EMVs for 6 h. **b** MDA content in H9c2 cells were determined after being treated with 10, 30, 60 μg/mL H/R-EMVs for 6 h. **c**, **d** Representative images and flow cytometry analysis of ROS production in H9c2 cells after H/R-EMVs treatment. ^**^ *p* < 0.01, ^***^ *p* < 0.001 versus Control; ^#^ *p* < 0.05, ^##^ *p* < 0.01, ^###^ *p* < 0.001 versus H/R-EMVs 10 μg/mL; ^&&&^ *p* < 0.001 versus H/R-EMVs 30 μg/mL

It has been reported that excessive generation of ROS plays a major role in the initiation of apoptosis during acute myocardial infarction. Specific ROS such as H_2_O_2_ or superoxide have been implicated as crucial mediators of apoptotic cell death [16]. As mentioned above, H/R-EMVs were confirmed to be pro-apoptotic, next we investigated whether H/R-EMVs induce excessive production of lipid peroxidation and ROS in target cardiomyocytes. Results showed that SOD (an eliminator of free radicals) activity of H9c2 cells decreased, while MDA (an indicator of lipid peroxidation) content increased markedly in 60 μg/mL H/R-EMVs-treated group when compared with control (*P* < 0.01, Fig. 4a, b). In further experiments, ROS production was examined by fluorescent microscopy and flow cytometry in DCFH-DA-labelled H9c2 cardiomyocytes. It was observed that ROS gradually accumulated in H9c2 cells with the increasing dose of H/R-EMVs (Fig. 4c). Flow cytometry analysis showed that DCF fluorescence (ROS level) in 60 μg/mL H/R-EMVs-treated group increased by 9.59 % compared with control (*P* < 0.01, Fig. 4d). These results suggested that H/R-EMVs trigger ROS production to induce target cell apoptosis and oxidative damage.

### H/R-EMVs up-regulated p-p38 MAPK and p-JNK1/2 expression in target H9c2 cardiomyocytes

**Fig. 5:**
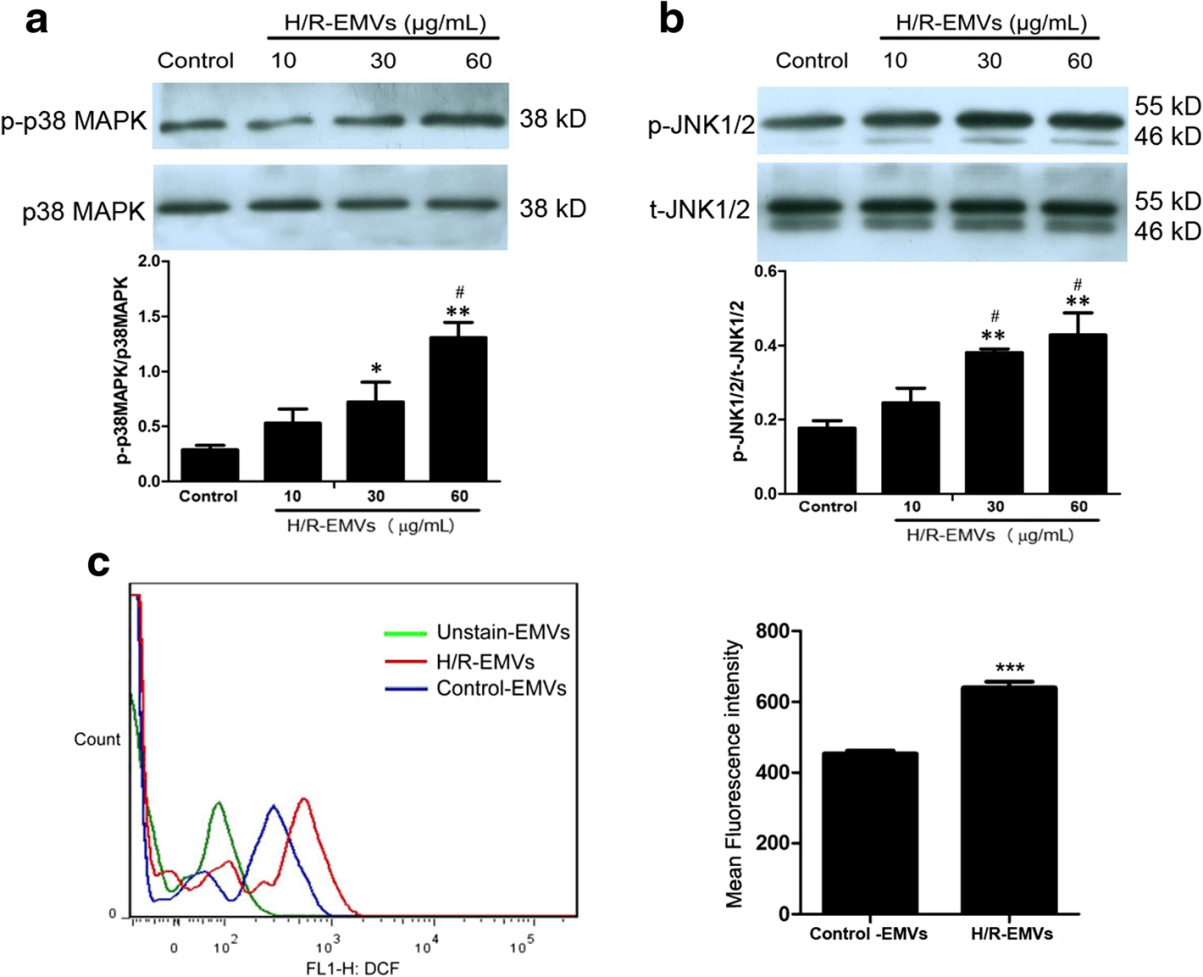
H/R-EMVs up-regulated the expression of p38 MAPK and JNK1/2 in H9c2 cells. **a** Representative images of immunoblots of p38 MAPK activation in each group. **b** JNK1/2 activation in H9c2 cells (phospho-JNK1/2). ^*^ *p* < 0.05, ^**^ *p* < 0.01 versus Control; ^#^ *p* < 0.05 versus H/R-EMVs 10 μg/mL. **c** ROS content in Control-EMVs and H/R-EMVs. ^***^ *p* < 0.001 versus Control-EMVs

In this study, we performed Western blot analysis on p38 and JNK1/2 activation of target H9c2 cells. The activation of p38 MAPK pathway was indicated by a significant increase of p38 phosphorylation in H9c2 cardiomyocytes treated with 30 or 60 μg/mL H/R-EMVs (*P* < 0.01, Fig. 5a). Moreover, the phosphorylation of JNK1/2 in H9c2 cells was up-regulated significantly in 30 and 60 μg/mL H/R-EMVs groups compared with control (*P* < 0.01, Fig. 5b). And the levels of p-p38 and p-JNK1/2 were increased in target cells in a dose-dependent manner. Thus, these findings showed that exposure of H9c2 cardiomyocytes to high dose H/R-EMVs results in activation of both the p38 and JNK1/2 signaling pathways.

### H/R-EMVs carried ROS

To determine which factor in H/R-EMVs might be responsible for p38 and JNK1/2 activation in H9c2 cardiomyocytes, we assessed ROS content in H/R-EMVs. EMVs derived from normal cultured HUVECs (without hypoxia12 h/reoxygenation 4 h) were introduced as control, which was defined as C-EMVs. Flow cytometry analysis showed that the DCF intensity of H/R-EMVs was significantly stronger than that of C-EMVs, which indicated that H/R-EMVs contained more ROS than C-EMVs (639.83 ± 41.03 v.s 453.67 ± 20.42, *P* < 0.001, Fig. 5c). Therefore, H/R-treated HUVECs released EMVs containing ROS. The ROS-containing EMVs might contribute to ROS production and subsequent oxidative stress in H9c2 cells.

## Discussion

IHD is a fatal disease and characterized by deficiency of coronary blood supply and impaired myocardium. MVs from various cellular origins have been identified in IHD, specially, EMVs account for a large proportion [17]. Accumulating evidences demonstrate that the elevated EMVs can actively modulate disease progression mostly including atherosclerosis and myocardial infarction [11]. EMVs could be interpreted as a vascular injury marker with prognostic value [18]. However, it is technically difficult to separate MVs from different origins in circulating blood. MVs are present at relatively low concentrations in normal physiological condition, but their levels increase in pathophysiological states [19, 20]. Recent evidences showed that MVs could be generated abundantly from cells undergoing inflammation, radiation, oxidative stress and so on [6]. We have reported that EMVs could be generated from cultured endothelial cells by calcium ionophore A23187 in the manner of magnificent calcium influx [21]. As reperfusion therapy plays a critical role in the treatment of IHD, further exploration on the mechanism of IRI is very necessary. In our research, treatments of HUVECs by H/R in vitro were used as a new approach to mimic IRI in vivo to generate EMVs. And flow cytometry analysis confirmed that the vesicles induced by H/R injury were EMVs with CD144 positive. However, MVs generated in normal physiological condition were hard to detect. Therefore, H/R-EMVs in different doses were used for functional studies.

Endothelial dysfunction is involved in the initial and core processes of the pathogenesis of IHD [22]. EMVs, released in response to endothelial cell activation or apoptosis, are significantly increased in patients with IHD, but their potential effect on myocardium is largely unknown. It has been reported that the level of EMVs among patients with myocardial infarction was positively correlated with the extent of vascular inflammation and myocardial infarct size [23]. EMVs generated from starved endothelial cells could dose-dependently suppress the endothelial cell proliferation with the dosage of 10^3^-10^5^ EMVs/mL [24]. Interestingly our results showed that high concentrations of H/R-EMVs could significantly promote cell apoptosis.

Cardiomyocyte apoptosis is a major event in the pathogenesis of IRI. Preliminary experiments indicated that in vivo myocardial I/R treatment produced obvious myocardial infarction, and TUNEL staining showed many apoptotic myocytes in the ischemic area [25]. Apoptosis is caused by an imbalance between pro-apoptotic and anti-apoptotic signals. It has already been known that Bax, Bcl-2, and caspase-3 are downstream molecules in mitochondrial apoptotic signaling pathway [26]. In particular, activation of caspase-3 plays a central role in the initiation of apoptosis. In our study, we found that the expression ratio of Bcl-2/Bax in target cardiomyocytes was down- regulated by H/R-EMVs but the activity of caspase 3 was enhanced. These results confirmed the pro-apoptotic effects of H/R-EMVs on cardiomyocytes in vitro.

Specific ROS have been implicated as crucial mediators of apoptotic cell death. EMVs exposed to AT1-AA (Angiotensin II receptor type 1 autoantibody) or high glucose condition all greatly increased ROS production in target cells. These “injured” EMVs trigged oxidative stress and induced endothelial dysfunction [27]. In agreement with these studies, our research found that H/R-EMVs increased ROS production in terms of increasing MDA content and decreasing SOD activity in H9c2 cells.

Because of their potential relevance to cell apoptosis and oxidative damage, we aimed to determine the possible pathway in which H/R-EMVs may participate. Multiple mechanisms have been proposed to explain myocardial injury during IRI. ROS lead to cell damage either directly or through behaving as intermediates in p38 MAPK and JNK1/2 downstream signaling pathways [28]. As expected, our study found that p38 and JNK1/2 were activated after treatment of 30 or 60 μg/mL H/R-EMVs. Additionally, it has been demonstrated that specific inhibition of p38 pathway resulted in reduced monocyte adhesion, in accordance with the down-regulation of ICAM-1 and VCAM-1 in target cells [14]. The use of ROS inhibitors could abolish EMVs-induced ROS production and reduce p38 phosphorylation in target cells. Moreover, addition of “injured” MVs to primary hepatocytes induced up-regulation of pro-inflammatory COX-2 and PKC-δ protein and the activation of JNK1/2 [13]. Taken together, these findings help us speculate that the activation of p38 and JNK1/2 could be triggered by ROS accumulation, suggesting that H/R- EMVs should probably promote oxidative stress in H9c2 cardiomyocytes through p38 MAPK and JNK1/2 pathways.

IRI induces oxidative stress and intense inflammatory response resulting from the capacity of endogenous constituents. Recently, the bioactive contents carried by MVs are of great concerns. It has been discovered that MVs are not merely debris; they can carry cytokines and nuclear materials such as DNA, RNA, and microRNA from their metrocyte [7–9]. MVs from endothelial progenitor cells could transfer mRNA to endothelial cells and activate an angiogenic program [9]. Here, we found H/R-EMVs carry ROS with significantly high levels, indicating ROS content in H/R-EMVs might have a link with oxidative stress in target cells, as well as the increasing ROS production. However, the exact mechanism of the increased ROS production in target cells still needs further investigation.

## Conclusion

In this study, we first established that EMVs could be generated from H/R-treated HUVECs. Then we demonstrated that 60 μg/mL H/R-EMVs exerted pro-apoptotic and oxidative effects on H9c2 cardiomyocytes via p38 and JNK1/2 signaling pathways. ROS carried by H/R-EMVs might be the underlying pathway to explicate their roles in apoptosis and oxidative stress. These findings indicated that the connection of EMVs and cardiomyocyte death would be interpreted as a novel intervention to study IRI, suggesting that decreasing the levels of EMVs should be a new therapeutic strategy for the maintenance of endothelial homeostasis and the treatment of IHD. However, whether other bioactive molecules in EMVs are contributable to myocardial injury is not clear. Moreover, our results need to be confirmed with the study of myocardial I/R models in vivo.

### Ethics

All procedures were performed in accordance with the Declaration of Helsinki of the World Medical Association and the research protocol was approved by Ethics Committee of Tianjin Medical University.

### Consent

All authors have read and approved the submission of this manuscript.

## Abbreviations

DCFH-DA: 2′,7′-dichlorodihydrofluorescein diacetate
HUVECs: human umbilical vein endothelial cells
H/R: hypoxia/reoxygenation
IHD: ischemic heart disease
IRI: ischemia/reperfusion injury
LDH: lactate dehydrogenase
MDA: malondialdehvde
MTT: methyl thiazolyl tetrazolium
MVs: microvesicles
PFA: paraformaldehyde
ROS: reactive oxygen species
RT: room temperature
SOD: superoxide dismutase
TBARS: thiobarbituric acid reactive substance
